# The efficacy and safety of cardio-protective therapy in patients with 5-FU (Fluorouracil)-associated coronary vasospasm

**DOI:** 10.1371/journal.pone.0265767

**Published:** 2022-04-07

**Authors:** Amna Zafar, Zsofia D. Drobni, Matthew Lei, Carlos A. Gongora, Thiago Quinaglia, Uvette Y. Lou, Ramya Mosarla, Sean P. Murphy, Maeve Jones-O’Connor, Ali Mahmood, Sarah Hartmann, Hannah K. Gilman, Colin D. Weekes, Ryan Nipp, John R. Clark, Jeffrey W. Clark, Lawrence S. Blaszkowsky, Erica Tavares, Tomas G. Neilan

**Affiliations:** 1 Department of Radiology and Division of Cardiology, Cardiovascular Imaging Research Center (CIRC), Massachusetts General Hospital, Harvard Medical School, Boston, Massachusetts, United States of America; 2 Division of Cardiovascular Diseases and Hypertension, Department of Medicine, Robert Wood Johnson University Hospital, Rutgers Medical School, New Brunswick, New Jersey, United States of America; 3 Cardiovascular Imaging Research Group, Heart and Vascular Center, Semmelweis University, Budapest, Hungary; 4 Division of Oncology and Hematology, Department of Medicine, Massachusetts General Hospital, Harvard Medical School, Boston, Massachusetts, United States of America; 5 Department of Medicine, Massachusetts General Hospital, Harvard Medical School, Boston, Massachusetts, United States of America; 6 Division of Cardiology, Department of Medicine, Morristown Medical Center, Morristown, New Jersey, United States of America; 7 Division of Cardiology, Department of Medicine, Cardio-Oncology Program, Massachusetts General Hospital, Harvard Medical School, Boston, Massachusetts, United States of America; Osaka University Graduate School of Medicine, JAPAN

## Abstract

**Background:**

Coronary vasospasm is a known side effect of 5-FU (fluorouracil) therapy. Beyond switching to non-5FU-based chemotherapy, there are no established treatments for 5-FU associated coronary vasospam. Our objective was to assess the safety and efficacy of re-challenge with 5-FU after pre-treatment with calcium channel blockers (CCBs) and long-acting nitrates among patients 5-FU associated coronary vasospasm.

**Methods:**

We conducted a retrospective study of patients with 5-FU coronary vasospasm at a single academic center. By protocol, those referred to cardio-oncology received pre-treatment with either combination [nitrates and CCBs] or single-agent therapy [nitrates or CCBs]) prior to re-challenge with 5-FU. Our primary outcome was overall survival. Other important outcomes included progression-free survival and safety.

**Results:**

Among 6,606 patients who received 5-FU from January 2001 to Dec 2020, 115 (1.74%) developed coronary vasospasm. Of these 115 patients, 81 patients continued 5-FU therapy, while 34 stopped. Of the 81 who continued, 78 were referred to cardio-oncology and prescribed CCBs and/or nitrates prior to subsequent 5-FU, while the remaining 3 continued 5-FU without cardiac pre-treatment. Of the 78, 56.4% (44/78) received both nitrates and CCBs, 19.2% (15/78) received CCBs alone, and 24.4% (19/78) received nitrates alone. When compared to patients who stopped 5-FU, those who continued 5-FU after pre-treatment (single or combination therapy) had a decreased risk of death (HR 0.42, P = 0.005 [95% CI 0.23–0.77]) and a trend towards decreased cancer progression (HR 0.60, P = 0.08 [95% CI 0.34–1.06]). No patient in the pre-treatment group had a myocardial infarct after re-challenge; however, chest pain (without myocardial infarction) recurred in 19.2% (15/78) among those who received cardiac pre-treatment vs. 66.7% (2/3) among those who did not (P = 0.048). There was no difference in efficacy or the recurrence of vasospasm among patients who received pre-treatment with a single agent (nitrates or CCBs) or combination therapy (14.7% (5/34) vs. 25.0% (11/44), P = 0.26).

**Conclusion:**

Re-challenge after pre-treatment with CCBs and nitrates guided by a cardio-oncology service was safe and allowed continued 5-FU therapy.

## Introduction

5-FLUOROURACIL (5-FU) is an antimetabolite that is a common standard therapy for several cancers, including adenocarcinomas and squamous cell carcinomas of the bladder, gastrointestinal tract, and head and neck [1]. However, use of 5-FU therapy may be limited due to the development of cardiotoxicities. There are several reported presentations of 5-FU associated cardiotoxicity; however, coronary artery vasospasm presenting as chest pain is the most common and described cardiotoxicity [2, 3]. Other less common presentations include arrhythmias, pericarditis, myocarditis, heart failure and even death [2–4]. The reported incidence of 5-FU cardiotoxicity in the literature varies from 1% to 35%; likely due to the sample population being studied, the inclusion of multiple different cardiac toxicities, and the varied formulations/administration protocols for the drug [2, 5].

The incidence of recurrent cardiotoxicity with 5-FU rechallenge without any adjustment in treatment is 90% [6, 7]. Therefore, there are several potential approaches once a patient is diagnosed with 5-FU vasospasm; switch the cancer treatment to a non-5-FU approach, switch to a bolus 5-FU approach, consider cardio-protective medications, or some combination of these approaches. The cardio-protective medication strategy for 5-FU vasospasm involves the use of nitrates and/or calcium channel blockers (CCBs), mirroring, without data to support it, the ACC/AHA guidelines for vasospastic angina [6, 8]. Nitrates and CCBs provide symptomatic relief of chest pain symptoms in the acute setting; however, universal pre-treatment with vasodilators has not been shown to decrease the risk of coronary vasospasm and is therefore not routinely recommended [9, 10]. We aimed to add to the limited data on the efficacy and safety of pre-treatment with nitrates and/or CCBs among patients with 5-FU vasospasm.

## Patients and methods

### Study design

We conducted a retrospective analysis of all patients who received 5-FU at a single academic center (Massachusetts General Hospital, Boston, Massachusetts) from January 2001 to December 2020. Individuals were flagged based on keyword search for ‘5-fluorouracil’ in their charts using RPDR (Research Patient Data Registry). 6,606 individual patients were identified from this search. The results were narrowed based on keyword search for ‘5-fluorouracil’ and ‘vasospasm’ among these individuals. Vasospasm was defined as the new occurrence of a typical chest pain syndrome at rest in the presence of recent 5-FU with or without ECG or biomarker changes. We further searched the list of 6,606 patients for the diagnosis of myocardial infarction based on ICD (International Classification of Diseases) codes, entered within a year of receiving 5-FU, to ensure completeness of our dataset. With this combined approach, 115 patients with vasospasm were identified. The diagnosis of 5-FU associated coronary vasospasm was independently adjudicated by two cardiologists. Demographics, baseline medical history and last known follow-up or date of death were collected by manual chart review from electronic medical records. Baseline parameters were identified from the oncology consult note prior to 5-FU initiation. Data regarding 5-FU dosing were obtained from pharmacy dispense records. The institutional review board approved the study, and the requirement for written informed consent was waived.

**Fig 1:**
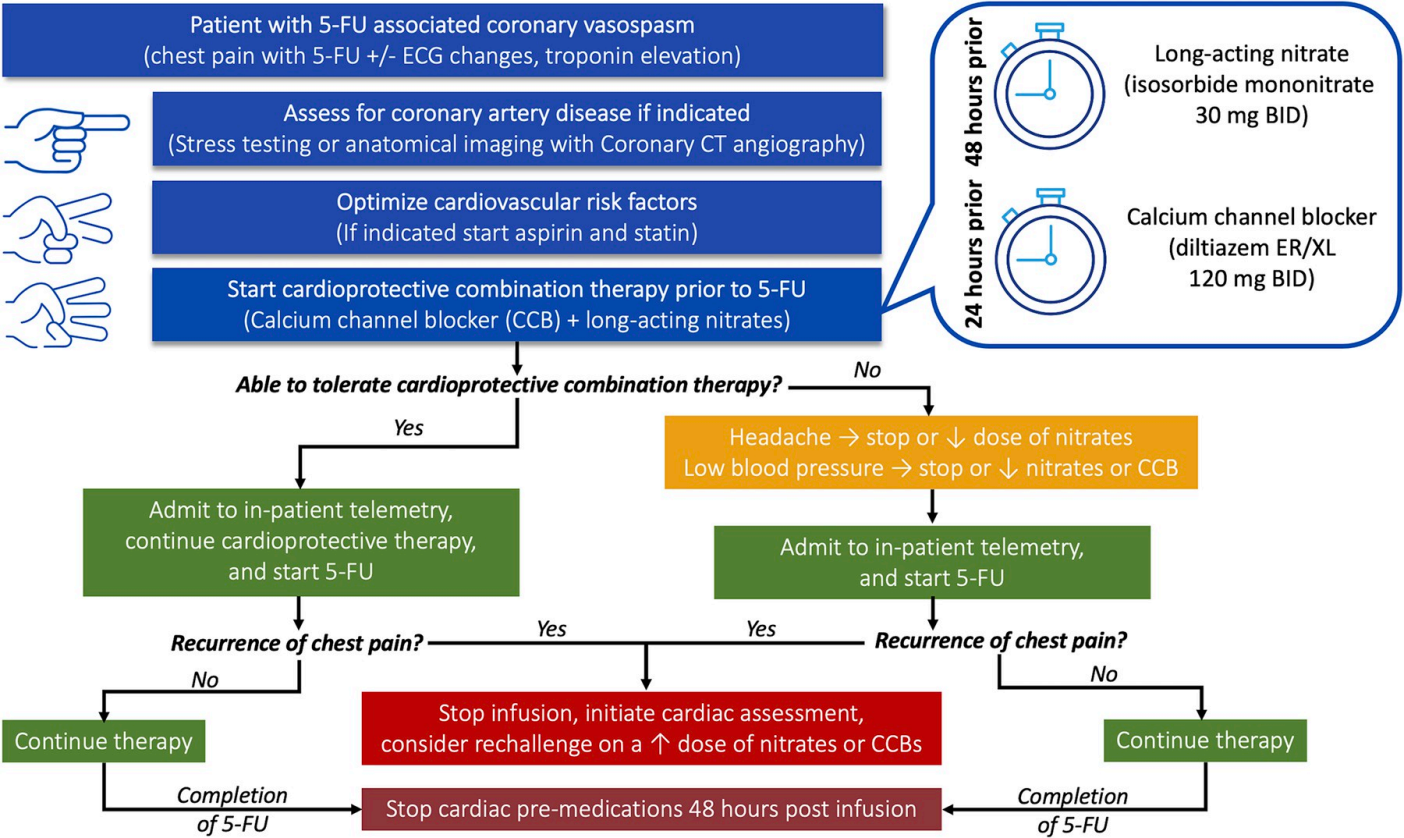
Proposed treatment algorithm for 5-FU (Fluorouracil) coronary vasospasm patients. Suggested treatment algorithm followed by cardio-oncology specialists at our institution.

Referral to cardio-oncology was based on provider preference (Fig 1). Once patients were referred, they were assessed for coronary artery disease (if indicated) using stress testing or anatomical imaging (coronary computed tomography angiography ((CCTA). Furthermore, their baseline cardiovascular disease risk factors were optimized by starting medical therapy as indicated (aspirin, and/or statin). Patients were then started on combination therapy consisting of long-acting nitrates (typically isosorbide mononitrate 30 mg twice daily [BID]) 48 hours prior and a CCB (typically diltiazem ER/XL 120 mg BID) 24 hours prior to rechallenge with 5-FU therapy. Of note, patients receiving nitrates and/or CCBs prior to development of vasospasm had their doses up titrated and/or had the missing agent added. If patients were able to tolerate combination therapy, they were admitted to an inpatient telemetry unit, and started on 5-FU therapy under the care of oncologists and cardio-oncologists. If patient specific factors limited use of combination therapy, such as headaches limiting nitrate use or hypotension/bradycardia limiting CCB use, then the dose of the culprit medication was adjusted to tolerance, followed by in-patient admission for 5-FU rechallenge. Patients received at least 2–3 cycles of therapy with inpatient monitoring. If chest pain did not recur during those admissions, patients continued outpatient 5-FU therapy along with cardio-protective medications. However, if chest pain recurred, rechallenge with higher doses of cardio-protective medications was only considered after a detailed discussion with the patient, oncologist and cardio-oncologist. Cardio-protective medications were continued for 48 hours after completion of 5-FU therapy during each cycle.

### Statistical analyses

Baseline characteristics are presented as continuous variables summarized as mean ± standard deviation (SD), or categorical variables summarized as counts with percentages (%). Differences between continuous variables were assessed using the t-test, and between categorical variables using the chi-square test. Overall survival (OS) and progression free survival (PFS) between the two groups were analyzed using Kaplan-Meier curves and log-rank test. A multivariable variable Cox-regression model was constructed to adjust for baseline variables that were differentially distributed between the two groups. All statistical tests were 2-sided and 5% was set as the level of significance. Statistical analysis was performed using STATA version 15.1 (StataCorp, College Station, Texas).

## Results

### Patient characteristics

**Fig 2:**
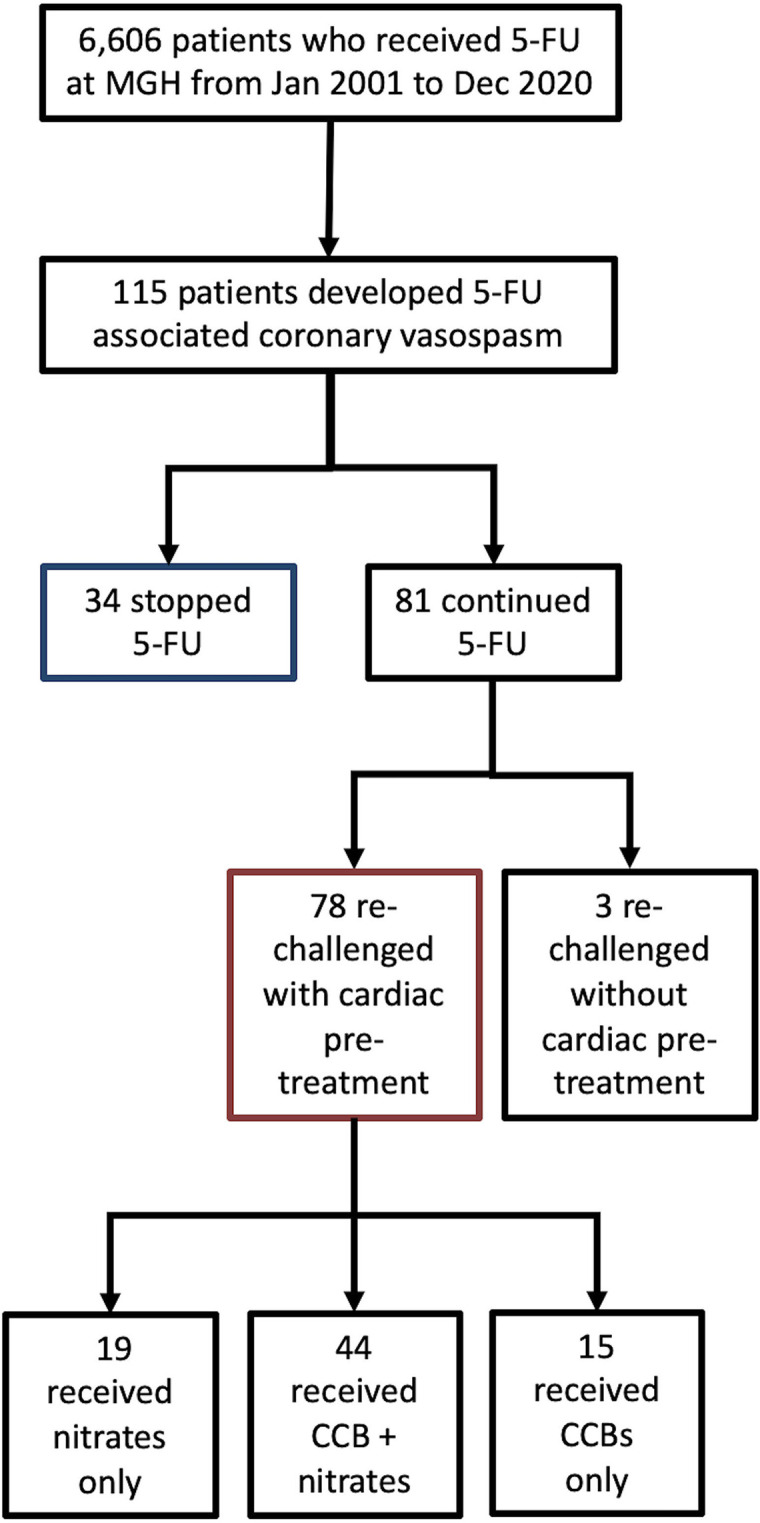
Summary of treatment assignments for 5-FU (Fluorouracil) associated coronary vasospasm patients. Summary of treatment assignments for all patients who developed 5-FU (Fluorouracil) associated coronary vasospasm.

Among 6,606 individual patients who received 5-FU, 115 patients (1.74%) developed 5-FU associated coronary vasospasm. Overall 81 patients were re-challenged with 5-FU therapy, while 34 stopped 5-FU (Fig 2). Among these 81, 3 were re-challenged without cardiac pre-treatment (S1 Table), while 78 patients were referred to cardio-oncology and received cardiac pre-medications prior to re-challenge. Of the 78 patients, 56.4% (44/78) received combination therapy with long-acting nitrates and CCBs, 24.4% (19/78) received long-acting nitrates only, and 19.2% (15/78) received CCBs only.

Baseline demographics, cancer and medical histories, and 5-FU regimens of all vasospasm patients are outlined in Table 1. Age, gender, race, stage of cancer, and baseline medication use between patients who were re-challenged with 5-FU therapy and those who stopped were similar. There was a higher rate of upper GI cancers among patients who stopped 5-FU therapy (35.3% vs. 10.3%, P = 0.001), while there was a higher proportion of colorectal cancers among patients who were re-challenged with 5-FU therapy (65.4% vs. 32.4%, P = 0.001). Similarly, there was a higher proportion of current or prior smoking (64.7% vs. 33.3%, P = 0.002) among those who stopped 5-FU therapy.

**Table 1:** Baseline characteristics of all patients with vasospasm, and comparison of those of who continued 5-FU therapy with cardiac pre-treatment vs. those who stopped 5-FU therapy.

	All Patients with Vasospasm (N = 115)*	Patients with Pre-treatment (N = 78)*	Patients who Stopped 5-FU (N = 34)*	P value
**Demographics**				
Age	58±12	56±12	61±12	0.05
Females (%)	66 (57.4)	41 (52.7)	24 (70.6)	0.08
White (%)	101 (87.8)	71 (91.0)	28 (82.4)	0.19
**Oncology History**				
Cancer stage				
• Stage I	8 (7.0)	5 (6.4)	3 (8.8)	0.65
• Stage II	15 (13.0)	10 (12.8)	4 (11.8)	0.89
• Stage III	46 (40.0)	32 (41.0)	13 (38.2)	0.78
• Stage IV	46 (40.0)	31 (39.7)	14 (41.2)	0.89
Upper gastrointestinal cancer (%)	20 (17.4)	8 (10.3)	12 (35.3)	**0.001**
Colorectal cancer (%)	64 (55.7)	51 (65.4)	11 (32.4)	**0.001**
Pancreatic cancer (%)	20 (17.4)	13 (16.7)	6 (17.7)	0.90
Other cancer (%)	11 (9.6)	6 (7.7)	5 (14.7)	0.25
**Medical History**				
Hypertension	56 (48.7)	34 (43.6)	21 (61.8)	0.08
Hyperlipidemia	52 (45.2)	29 (37.2)	22 (64.7)	**0.007**
Diabetes mellitus	24 (20.9)	13 (16.7)	11 (32.4)	0.06
Smoking	50 (43.5)	26 (33.3)	22 (64.7)	**0.002**
Ischemic heart disease	20 (17.4)	14 (18.0)	6 (17.7)	0.97
Chronic kidney disease	9 (7.9)	5 (6.5)	3 (8.8)	0.66
**Baseline Medications**				
ASA	34 (29.6)	23 (29.5)	10 (29.4)	0.99
Beta blockers	32 (27.8)	21 (26.9)	11 (32.4)	0.56
ACE-i/ARB	38 (33.3)	22 (28.6)	15 (44.1)	0.11
Aldosterone antagonist	4 (3.5)	3 (3.9)	1 (2.9)	0.81
Nitrate	8 (7.0)	7 (9.0)	1 (2.9)	0.25
Calcium channel blockers	11 (9.6)	5 (6.4)	6 (17.7)	0.07
• Dihydropyridine	6 (5.2)	2 (2.6)	4 (11.8)	**0.047**
• Non-dihydropyridine	5 (4.3)	3 (3.8)	2 (5.9)	0.23
**5-FU Regimens Combination (bolus and infusion) Regimens**				
FOLFIRINOX (5-FU + leucovorin + irinotecan + oxaliplatin)	18 (15.7)	13 (16.7)	5 (14.7)	N/A
FOLFIRI (5-FU + leucovorin + irinotecan)	4 (3.5)	4 (5.1)	0 (0)	N/A
FOLFIRI + cetuximab	2 (1.7)	0 (0)	1 (2.9)	N/A
FOLFOX (5-FU + leucovorin + oxaliplatin)	50 (43.5)	35 (44.9)	13 (38.2)	N/A
FOLFOX + bevacizumab or traztuzumab	5 (4.3)	5 (6.4)	0 (0)	N/A
5FU + leucovorin	1 (0.9)	0 (0)	1 (2.9)	N/A
5FU + irinotecan	1 (0.9)	1 (1.3)	0 (0)	N/A
TPF (5-FU + docetaxel + cisplatin)	3 (2.6)	1 (1.3)	2 (5.9)	N/A
**Total**	84 (73.9)	59 (75.6)	22 (64.7)	
**Bolus Only Regimens**				
FLOX (5-FU + leucovorin + oxaliplatin)	1 (0.9)	0 (0)	1 (2.9)	N/A
5-FU IV bolus only	1 (0.9)	0 (0)	1 (2.9)	N/A
5-FU + cyclophosphamide + methotrexate (CMF)	1 (0.9)	1 (1.3)	0 (0)	N/A
**Total**	3 (2.6)	1 (1.3)	2 (5.9)	
**Infusion Only Regimens**				
5-FU IV infusion	8 (7.0)	5 (6.4)	3 (8.8)	N/A
5-FU IV infusion + radiation	3 (2.6)	2 (2.6)	1 (2.9)	N/A
5-FU IV infusion + erlotinib + bevacizumab	1 (0.9)	1 (1.3)	0 (0)	N/A
FOLFOXIRI (5-FU + leucovorin + irinotecan + oxaliplatin)	2 (1.7)	2 (2.6)	0 (0)	N/A
FOLFOXIRI + bevacizumab	1 (0.9)	1 (1.3)	0 (0)	N/A
5FU + mitomycin	5 (4.3)	2 (2.6)	3 (8.8)	N/A
5FU + cisplatin	1 (0.9)	0 (0)	1 (2.9)	N/A
FLOT (5FU + docetaxel + oxaliplatin + leucovorin)	1 (0.9)	0 (0)	1 (2.9)	N/A
**Total**	22 (19.1)	13 (16.7)	9 (26.5)	
**Other**				
Capecitabine	6 (5.2)	5 (6.4)	1 (2.9)	N/A

Baseline characteristics, medical history and medication use of patients who developed vasospasm (N = 115), and comparison of patients who continued 5-FU (Fluorouracil) with cardiac pre-treatment (N = 78) versus those who stopped 5-FU therapy (N = 34) after development of vasospasm. *Not shown are 3 patients from total of 115, who continued 5-FU therapy without cardiac pre-treatment (S1 Table). Continuous variables are presented as the mean±standard deviation, and categorical variables are represented with their absolute counts and percents. P values were calculated using t-test for continuous variables and chi square test for categorical variables, when comparing patients who continued 5-FU therapy after pre-treatment (N = 78) and those who stopped 5-FU therapy (N = 34). Of note, P values were not generated when the sample sizes were too small to reflect meaningful differences between the groups, as in the case of different formulations of 5-FU therapy.

### Oncologic efficacy

**Fig 3:**
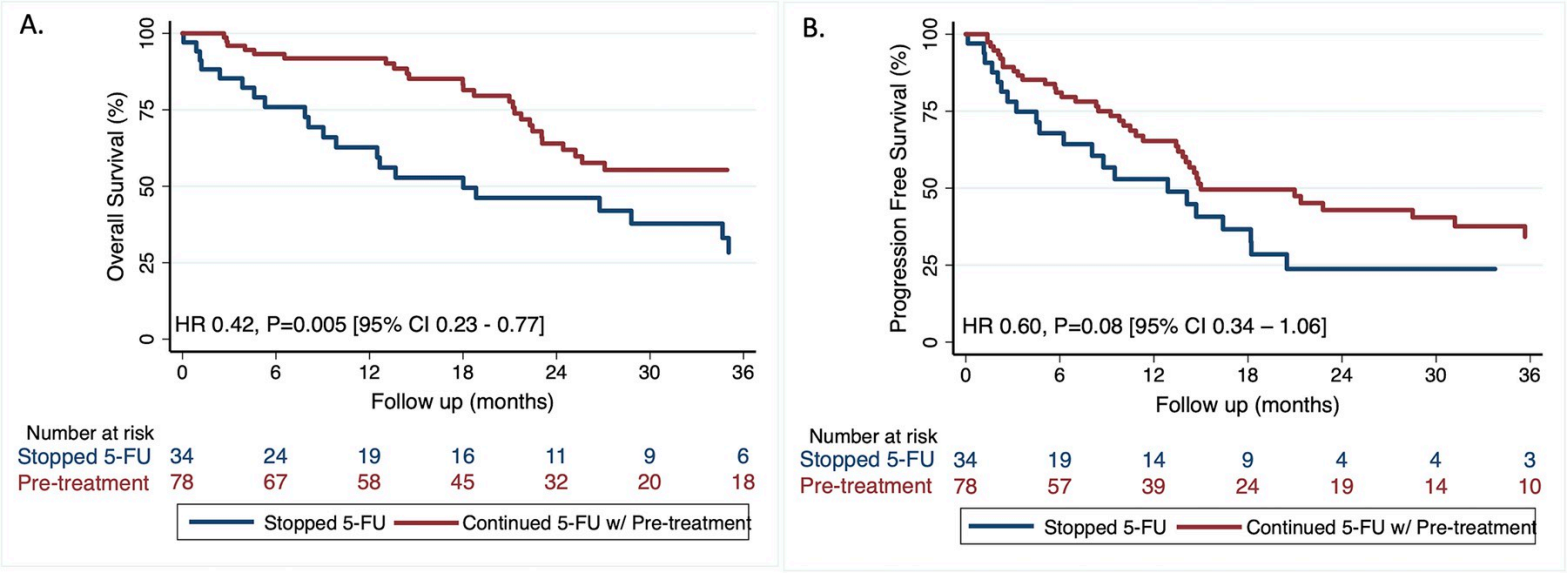
Kaplan-Meier curves for overall survival and progression free survival for 5-FU associated coronary vasospasm patients. Comparison of (A) overall survival and (B) progression free survival in patients who developed 5-FU associated coronary vasospasm and continued 5-FU therapy after receiving cardioprotective pre-treatment compared to those who stopped 5-FU therapy.

The median overall survival (OS) for patients who continued 5-FU with cardiac pre-medications was 47 months (95% CI 23.4 –NR months) as compared to 18.3 months (95% CI 9.2–35.5 months) for those who stopped 5-FU (P = 0.003) (Fig 3A). Similarly, median progress-free survival (PFS) for patients who continued 5-FU with cardiac pre-medications was 15.2 (95% CI 13.7–36.2) months as compared to 13.1 (95% CI 4.8–18.5) months for those who stopped 5-FU (P = 0.07) (Fig 3B). As compared to patients who stopped 5-FU, those who continued after pre-treatment (single or combination therapy) had a decreased risk of death (HR 0.42, P = 0.005 [95% CI 0.23–0.77]) and a trend towards decreased cancer progression (HR 0.60, P = 0.08 [95% CI 0.34–1.06]), after adjustment for age, initial stage of cancer, smoking, colorectal and upper gastrointestinal cancers.

### Cardiac safety and efficacy

Chest pain (without myocardial infarction) recurred in 19.2% (15/78) among those who received cardiac pre-treatment vs. 66.7% (2/3) among those who did not (P = 0.048). Among the 63/78 patients who successfully tolerated additional 5-FU therapy with cardiac pre-medications, 9 patients were also transitioned to bolus only regimens. There was no difference in the recurrence of vasospasm symptoms among patients who received pre-treatment with a single agent (long-acting nitrates or CCBs) or combination therapy, 14.7% (5/34) vs. 25.0% (11/44), P = 0.26. Furthermore, among patients who received single agent pre-treatment, there was no difference in the recurrence of vasospasm symptoms among those who received long-acting nitrates or CCBs, 21.1% (4/19) vs. 6.7% (1/15), P = 0.24.

## Discussion

In this retrospective study of patients with 5-FU associated coronary vasospasm, we demonstrate the efficacy and safety of re-challenging patients after pre-treatment with cardio-protective medications. The median overall survival (OS) of patients who continued to receive 5-FU with cardiac pre-medications was longer by 28.7 months when compared to those who stopped therapy. Furthermore, patients who continued 5-FU therapy after pre-treatment had a decreased risk of death, when compared to those who stopped. Patients who received cardiac pre-treatment were also less likely to experience recurrent vasospasm compared to those who continued 5-FU without pre-treatment. This approach appeared safe as, of the recurrent vasospasm cases who were pre-treated with CCB and or long-acting nitrates, none had a myocardial infarction. As the reported incidence of recurrent cardiotoxicity with 5-FU rechallenge without changing the treatment approach is 90% [6, 7], our findings of an effective and safe re-challenge in this patient population have direct relevance to clinical care.

There are limited data on the successful re-challenge of 5-FU vasospasm patients with or without pre-treatment with anti-anginal therapies. Tsavaris et al. observed that ‘intensive cardiologic monitoring and prophylactic nitrate administration may result in fairly good subsequent tolerance,’ among the 20 patients who developed coronary vasospasm in their study [11]. Similarly, calcium channel blockers such as diltiazem have been used to treat, and re-challenge patients who developed vasospasm after their first 5-FU exposure in small case series [7, 12, 13]. However, prophylactic use of vasodilators in all patients is discouraged due to paucity of data and sometimes conflicting data; some prospective studies where patients were pre-treated prior to their first infusion, did not show any difference in the incidence of toxicity between the treatment and control groups [14]. Kwakman et al. successfully rechallenged 7 patients with oral fluoropyrimidine S-1, a therapy with a lower concentration of cardiotoxic metabolites than 5-FU or capecitabine, after these patients had coronary vasospasm with capecitabine [15]. There are minimal long-term oncologic outcome data on these patients that were either treated with lower doses of 5-FU or different formulations.

Our methodical approach to a cardiac evaluation and pre-treatment therapy (Fig 1) highlights the value of cardio-oncology input in these challenging cases. While outlining a clear mechanism for the improved OS is beyond the scope of our study, we hypothesize that longer OS in our patients who continued 5-FU therapy is due to the continued availability of this key cancer medication. Our study demonstrates the safety and efficacy of a pre-treatment approach, however, future prospective studies that randomize patients to re-challenge after cardiac pre-treatment versus changing or stopping 5-FU therapy will be needed to further validate this protocol.

### Study limitations

This was a retrospective study design; however, given the low incidence of coronary vasospasm amongst all comers receiving 5-FU therapy, a prospective design would not have been feasible. The relatively large scale of the study allowed us to show a statistically significant difference in overall survival between patients who were rechallenged and those who stopped 5-FU chemotherapy. Lastly, we acknowledge some potential selection bias when comparing patients who stopped 5-FU and those who continued 5-FU therapy after pre-treatment, since patients who stopped 5-FU therapy were possibly sicker than patients who were able to continue after pre-treatment. We attempted to address this limitation by controlling for confounders that were different between the two groups, namely age, initial stage of cancer, smoking, colorectal and upper gastrointestinal cancers. Furthermore, we were unable to directly compare patients who continued 5-FU therapy with and without pre-treatment due to the very small number of patients who continued 5-FU therapy without pre-treatment (only 3, additional details provided in S1 Table). Nonetheless, this remains a limitation of our study, and supports the need for studies where patients with 5-FU vasospasm are randomized to pre-treatment with calcium channel blockers and/or nitrates, or placebo [16].

## Conclusions

In the largest report of 5-FU-associated vasospasm, re-challenge after pre-treatment under the guidance of cardio-oncologists with CCBs and nitrates was safe and allowed continued 5-FU therapy, resulting in improved overall survival and a trend towards improved progression free survival.

## Supporting information

S1 Table Patients re-challenged with 5-FU without cardiac pre-treatment. Clinical follow-up information for patients who were re-challenged with 5-FU therapy without cardiac pre-treatment (calcium channel blockers and/or long-acting nitrates).
(DOCX)

S1 File (PDF)

## Abbreviations

5-FU: 5-Fluorouracil
ECG: Electrocardiogram
ACE-i: Angiotensin converting enzyme inhibitor
ARB: Angiotensin II receptor blocker
CCBs: Calcium channel blockers
RPDR: Research Patient Data Registry
ICD: International Classification of Diseases
