# Supplementary material for: The efficacy and safety of cardio-protective therapy in patients with 5-FU (Fluorouracil)-associated coronary vasospasm
Source: PLoS One. 2022 Apr 7;17(4):e0265767. doi: 10.1371/journal.pone.0265767 (PMC8989300; doi:10.1371/journal.pone.0265767)
Supplement: S1 Table — Clinical follow-up information for patients who were re-challenged with 5-FU therapy without cardiac pre-treatment (calcium channel blockers and/or long-acting nitrates). (DOCX) [file pone.0265767.s001.docx]

***S1 Table.* Patients Re-Challenged with 5-FU without Cardiac Pre-treatment**

| Patient #1 | Patient admitted and diagnosed with coronary vasospasm after fourth cycle; however, later reported chest pain symptoms that started with the first cycle. Patient did not receive any additional 5-FU after fourth cycle. |
| --- | --- |
| Patient #2 | Patient continued to experience chest pain which was the initial presenting complaint with 5-FU vasospasm but went on to complete 5-FU therapy. |
| Patient #3 | 5-FU therapy was changed from infusion to bolus only regimen. Patient tolerated 3 cycles of therapy post vasospasm. |
