# Supplementary material for: The efficacy and safety of cardio-protective therapy in patients with 5-FU (Fluorouracil)-associated coronary vasospasm
Source: PLoS One. 2022 Apr 7;17(4):e0265767. doi: 10.1371/journal.pone.0265767 (PMC8989300; doi:10.1371/journal.pone.0265767)
Supplement: S1 File — (PDF) [file pone.0265767.s002.pdf]

mrn	age_of_pres	days_follow_	smoking	htn_2	dm_2	hyperchol_2
315	46	1626	1	0	0	0
238	53	1027	1	0	0	1
311	71	726	1	1	1	1
306	65	34	1	1	1	1
90	75	416	1	0	0	1
250	80	275	1	1	0	1
213	68	876	1	1	0	1
224	51	1281	0	0	0	1
86	69	548	1	1	0	1
192	72	37	1	1	1	1
183	71	161	0	0	0	1
313	64	193	0	0	0	1
236	63	703	1	1	0	0
84	64	239	1	0	1	1
42	69	137	1	0	1	0
201	48	246	0	1	0	0
62	58	1054	0	0	1	1
314	73	2	0	1	1	1
195	55	117	1	1	0	0
317	63	73	1	1	0	1
231	80	385	0	1	0	0
184	63	380	1	1	1	1
211	66	140	1	1	1	1
235	50	617	1	1	0	0
221	42	1440	1	0	0	0
248	61	1271	0	1	0	1
335	25	27	0	0	0	0
149	72	300	1	1	1	1
325	51	2932	1	1	1	1
323	54	1066	1	0	0	0
105	50	3304	0	1	0	1
324	55	814	1	1	0	0
307	73	112	0	1	0	1
18	67	573	0	0	0	0
103	67	442	0	0	0	0
199	70	391	0	0	0	1
92	60	694	0	0	0	0
237	43	868	0	0	0	0
223	68	548	0	1	1	0
146	49	397	0	0	0	1
210	25	547	0	0	0	0
129	39	372	0	0	0	0
140	78	2180	0	0	0	0
229	44	1597	0	1	1	1

63	52	609	0	0	0	0
197	33	185	1	0	0	0
171	47	309	0	0	0	0
226	57	648	0	1	0	0
5	48	1002	0	0	0	0
186	46	447	0	0	0	0
180	46	310	0	0	0	0
242	51	638	0	0	0	1
245	80	757	0	1	0	1
1	51	787	1	1	0	1
239	51	787	1	1	0	0
189	48	329	0	0	0	0
309	61	33	0	1	0	0
252	53	1260	0	0	0	0
164	58	140	1	1	0	0
104	65	2562	0	1	0	1
308	63	67	0	0	0	0
246	57	81	0	0	0	0
310	46	384	1	0	0	0
240	58	895	0	0	1	0
318	51	181	1	1	0	1
225	49	3486	0	0	0	0
187	78	449	0	1	0	1
316	54	211	0	0	0	0
251	78	230	1	1	1	0
228	60	1920	1	1	1	1
193	48	780	1	0	0	1
94	64	2216	0	1	1	1
8	84	683	0	1	1	0
139	51	661	1	0	0	1
7	67	438	1	1	0	1
220	46	2292	1	1	0	1
217	34	824	0	0	0	0
222	72	3128	0	1	0	1
119	44	569	0	0	0	0
6	46	903	0	0	0	0
156	44	767	0	0	0	0
319	58	640	1	0	0	0
232	65	86	1	0	0	1
320	33	1410	1	1	0	1
102	68	1063	1	1	1	0
85	46	678	0	0	0	0
333	58	88	1	1	1	1
334	63	167	1	0	0	0
230	76	539	0	1	0	0

330	61	122	0	1	0	1
244	41	1792	0	1	0	0
249	64	6990	1	0	0	0
93	80	733	0	0	0	0
44	57	199	0	1	0	1
182	47	701	0	1	0	1
331	39	743	1	0	0	0
332	68	843	1	1	1	0
4	79	1521	1	1	0	1
194	51	645	0	0	0	0
20	58	2605	1	1	0	1
89	42	2039	0	1	0	0
215	66	1540	0	0	0	0
76	58	1277	0	1	0	0
322	67	413	0	1	1	1
321	67	1733	1	1	0	1
227	54	702	0	0	0	0
326	50	22	0	0	1	0
181	61	545	0	0	0	0
327	52	46	0	0	1	0
185	61	470	0	0	0	1
328	70	224	1	0	0	1
329	54	224	1	1	0	1
219	63	301	1	0	0	0
312	54	1114	1	1	0	1
134	66	1556	0	0	0	0

ihd	ckd	asa_baseline	bblocker_bas	ace_i_arb_ba	aldosterone_	ca_channel_
0	0	0	0	0	0	0
0	0	1	0	0	0	0
1	0	1	1	1	0	0
0	1	0	1	0	0	1
1	0	0	1	1	0	0
1	0	0	0	1	0	1
0	0	1	0	1	0	1
0	0	0	0	0	0	0
0	0	0	1	1	0	0
0	0	0	0	0	0	0
0	0	0	0	0	0	0
0	0	0	0	0	0	0
0	0	0	0	0	0	0
0	0	0	0	0	0	0
0	0	0	0	0	0	0
0	0	0	0	0	0	0
0	0	0	1	1	0	0
0	0	0	0	1	0	1
0	0	0	0	0	0	0
0	0	1	1	0	0	0
1	0	1	1	1	0	0
0	1	0	1	1	0	0
1	0	1	1	1	0	0
0	0	0	0	0	0	0
0	0	0	0	0	0	0
0	0	0	0	1	0	0
0	0	0	1	0	1	0
0	0	1	1	1	0	0
1	0	0	0	1	0	0
0	0	1	0	0	0	0
0	0	1	0	0	0	0
0	0	0	0	1	0	1
0	1	0	0	1	0	1
0	0	1	0	0	0	0
0	0	0	0	0	0	0
0	0	1	0	0	0	0
0	0	0	0	0	0	0
0	0	0	0	0	0	0
0	0	1	1	0	0	1
0	0	0	0	0	0	0
0	0	0	0	0	0	0
0	0	0	0	0	0	0
0	0	0	0	0	0	0
0	0	0	1	0	0	1

0	0	0	0	0	0	0
0	0	0	0	0	0	0
0	0	0	0	0	0	0
0	0	0	0	1	0	0
0	0	0	0	0	0	0
0	0	0	0	0	0	0
0	0	0	0	0	0	0
0	0	0	0	0	0	0
0	0	0	0	0	0	1
1	0	1	1	0	0	0
1	0	1	1	1	0	0
0	0	0	0	0	0	0
0	0	0	0	1	0	0
0	0	0	0	0	0	0
1	1	1	1	0	0	0
0	0	0	0	0	0	0
0	0	0	0	0	0	0
0	0	0	0	0	0	0
0	0	0	0	0	0	0
0	0	0	1	1	0	0
0	0	0	1	1	0	0
0	0	0	0	0	0	0
1	0	1	1	1	0	0
0	0	0	0	0	0	0
0	0	0	1	1	0	0
1	0	1	1	1	0	0
0	0	0	0	0	0	0
1	0	1	1	1	0	0
1	1	1	0	1	0	0
0	0	1	1	1	0	0
0	0	0	0	1	0	0
0	0	0	0	1	0	0
0	0	0	0	0	0	0
0	0	1	0	1	0	0
0	0	0	0	0	0	0
0	0	0	0	0	0	0
0	0	0	0	0	0	0
0	0	1	1	0	0	0
0	0	0	0		0	0
0	0	0	1	0	0	0
1	0	1	1	0	0	0
0	0	0	0	0	0	0
0	0	0	0	0	1	0
0	0	0	0	0	0	0
0	0	0	1	0	0	0

0	0	1	0	0	0	1
0	0	0	0	0	0	0
1	0	1	1	1	0	0
1	0	0	0	1	0	0
0	0	1	1	0	1	0
0	0	1	1	0	0	1
0	0	0	0	0	0	0
1	0	1	1	1	0	0
1	1	1	0	1	0	0
0	0	0	0	0	0	0
0	1	0	0	1	0	0
0	0	0	0	0	0	0
0	0	0	0	0	0	0
0	0	0	0	1	0	0
1	1	1	0	1	0	0
0	0	0	0	0	1	0
0	0	1	0	0	0	0
0	0	0	0	0	0	0
0	0	0	0	0	0	0
0	0	0	0	0	0	0
0		1	0	0	0	0
0	0	0	0	0	0	0
1	0	1	1	1	0	0
0	0	0	0	0	0	0
0	1	1	0	1	0	0
0	0	0	0	0	0	0

type_of_ccb	nitrate_base	treatment	type_of_trea	prog_of_ca	days_to_prog	death
	0	0		0	1626	0
	0	2		0	1027	0
	0	0		1	623	0
1	0	0		1	4	1
	0	0		1	137	1
2	0	0		1	51	1
1	0	2		1	554	1
	0	0		1	1096	1
	0	2		1	289	1
	0	2		1	37	1
	0	0		1	35	1
	0	0		0	193	0
	0	0		0	703	0
	0	0		1	143	1
	0	0		0	137	0
	0	0		1	98	1
	0	0		1	245	1
1	0	0		0	2	1
	0	0		1	81	1
	0	0		0	73	1
	1	0		0	385	1
	0	0		1	190	1
	0	2		1	62	1
	0	2		0	617	0
	0	0		0	1440	0
	0	0		1	447	1
	0	0		0	27	1
	0	0		1	69	1
	0	0		1	429	0
	0	0		1	267	1
	0	0		1	553	0
1	0	2		1	392	1
2	0	0		0	112	0
	0	0		1	499	1
	0	1	4	1	110	1
	0	1	2	1	67	0
	0	1	2	1	412	0
	0	1	2	0	868	0
2	0	1	2	1	257	1
	0	1	2	1	101	1
	0	1	3	1	175	1
	0	1	2	1	456	0
	0	1	3	1	949	0
1	0	1	3	1	693	0

	0	1	2	0	609	0
	0	1	2	0	185	0
	0	1	2	0	309	0
	0	1	4	1	319	1
	0	1	2	0	1002	0
	0	1	2	0	447	0
	0	1	2	0	310	0
	0	1	4	1	42	1
2	0	1	2	0	757	0
	1	1	2	1	253	0
	0	1	2	1	154	0
	0	1	2	0	329	0
	0	1	2	0	33	0
	0	1	4	1	443	0
	0	1	3	1	54	1
	0	1	4	1	344	0
	0	1	2	0	67	0
	0	1	3	1	42	1
	0	1	2	0	384	0
	0	1	3	0	895	0
	0	1	2	0	181	0
	0	1	2	1	1392	1
	0	1	2	0	449	0
	0	1	2	0	211	0
	0	1	2	0	230	0
	0	1	3	1	1654	0
	0	1	2	1	213	1
	0	1	4	0	2216	0
	0	1	3	1	48	1
	0	1	3	1	408	1
	0	1	2	1	434	1
	0	1	2	0	2292	0
	0	1	4	1	451	1
	0	1	3	0	3128	0
	0	1	3	1	298	1
	0	1	2	0	903	0
	1	1	2	1	186	1
	0	1	2	0	640	0
	0	1	3	0	86	1
	0	1	3	1	63	1
	0	1	3	0	1063	0
	0	1	4	1	421	1
	0	1	2	1	72	1
	0	1	4	0	167	0
	0	1	4	0	539	0

1	0	1	2	1	72	1
	0	1	4	0	1792	0
	1	1	2	0	6619	0
	0	1	3	1	448	0
	1	1	3	1	173	1
2	1	1	2	1	650	1
	0	1	3	1	306	1
	0	1	4	1	330	0
	1	1	2	0	1521	0
	0	1	4	1	638	1
	0	1	2	1	1085	0
	0	1	3	1	867	1
	0	1	3	0	1540	0
	0	1	4	1	427	1
	1	1	2	1	281	1
	0	1	2	1	1340	1
	0	1	2	1	93	1
	0	1	2	0	22	0
	0	1	2	0	545	0
	0	1	2	0	46	0
	0	1	2	0	470	0
	0	1	2	0	224	0
	0	1	4	0	224	0
	0	0		0	301	1
	0	0		0	1114	0
	0	0		1	84	1

cancer_deaths
1
1
1
1
1
1
1
1
1
1
1
1
1
1
1
0
1
1
1
1
1
1
1
1
1
1
1

1

1

1

1

1

1

1
1
1

1

1

1

1
1

1
1

1

1
1
1

1

1

1
1
1
1

0

1
